# Medical Society of Tennessee

**Published:** 1851-09

**Authors:** 


### MEDICAL SOCIETY OF TENNESSEE.

We have just received a copy of the Proceedings of the twenty-second annual meeting of the Medical Society of Tennessee, held at Murfreesborough, April, 1851, and find them replete with interesting matter, of which we shall avail ourselves in succeeding numbers. We have marked several of the cases reported and shall from time to time transfer them to our pages. Among them we were pleased to recognise a number from the pens of some of our valued correspondents. The Medieal Society of Tennessee is one of the most venerable of the scientific associations of the West, having now continued for nearly a quarter of a century. If it cannot boast of a very vigorous or very prosperous career, it has at least the credit of having out-lived many similar institutions; and it is no slight credit to the few members who have sustained it so long, that they rendered the last one of the most interesting of its many meetings. Y.

